# Review of Nitrification Monitoring and Control Strategies in Drinking Water System

**DOI:** 10.3390/ijerph19074003

**Published:** 2022-03-28

**Authors:** Sharif Hossain, Christopher W. K. Chow, David Cook, Emma Sawade, Guna A. Hewa

**Affiliations:** 1 Scarce Resources and Circular Economy (ScaRCE), UniSA STEM, University of South Australia, Mawson Lakes, SA 5095, Australia; christopher.chow@unisa.edu.au (C.W.K.C.); guna.hewa@unisa.edu.au (G.A.H.); 2 Future Industries Institute, University of South Australia, Mawson Lakes, SA 5095, Australia; 3 South Australian Water Corporation, Adelaide, SA 5000, Australia; david.cook@sawater.com.au (D.C.); emma.sawade@sawater.com.au (E.S.)

**Keywords:** rapid chloramine decay, nitrification, microbiological chloramine decay, nitrification monitoring and control, water disinfection, monochloramine

## Abstract

Nitrification is a major challenge in chloraminated drinking water systems, resulting in undesirable loss of disinfectant residual. Consequently, heterotrophic bacteria growth is increased, which adversely affects the water quality, causing taste, odour, and health issues. Regular monitoring of various water quality parameters at susceptible areas of the water distribution system (WDS) helps to detect nitrification at an earlier stage and allows sufficient time to take corrective actions to control it. Strategies to monitor nitrification in a WDS require conducting various microbiological tests or assessing surrogate parameters that are affected by microbiological activities. Additionally, microbial decay factor ($F_{m}$) is used by water utilities to monitor the status of nitrification. In contrast, approaches to manage nitrification in a WDS include controlling various factors that affect monochloramine decay rate and ammonium substrate availability, and that can inhibit nitrification. However, some of these control strategies may increase the regulated disinfection-by-products level, which may be a potential health concern. In this paper, various strategies to monitor and control nitrification in a WDS are critically examined. The key findings are: (i) the applicability of some methods require further validation using real WDS, as the original studies were conducted on laboratory or pilot systems; (ii) there is no linkage/formula found to relate the surrogate parameters to the concentration of nitrifying bacteria, which possibly improve nitrification monitoring performance; (iii) improved methods/monitoring tools are required to detect nitrification at an earlier stage; (iv) further studies are required to understand the effect of soluble microbial products on the change of surrogate parameters. Based on the current review, we recommend that the successful outcome using many of these methods is often site-specific, hence, water utilities should decide based on their regular experiences when considering economic and sustainability aspects.

## 1. Introduction

Drinking water treatment processes involve disinfecting water at the final stage of treatment to ensure water is microbiologically safe to consume. Usually, chlorine and chloramines are used to disinfect water followed by UV irradiation in some cases [1,2,3]. Although chlorine is a stronger disinfectant than chloramine, typically it is not suitable for long distribution systems where high-water age are encountered, as it decays faster than chloramine [1,3,4]. Disinfection using chlorine can also form substantially higher concentrations of regulated disinfection-by-products (DBPs) such as trihalomethanes (THMs) and haloacetic acids (HAAs) than does chloramine [1,5]. Using chloramine can ensure a relatively higher disinfection stability with less formation of regulated DBPs [1,3]. In a water distribution system (WDS), chloramine decay occurs via several pathways including chemical and microbiological processes [6]. A major concern in a chloraminated system is the occurrence of nitrification, where a significant reduction of chloramine residual occurs via microbiological decay process. Microorganisms, which are responsible for nitrification, mainly including ammonia-oxidising bacteria (AOB), ammonia-oxidising archaea (AOA), and nitrite-oxidising bacteria (NOB) [7,8,9,10,11,12]. Nitrification is a two-step microbiological process where, in the first stage, free ammonia resulting from chloramine decomposition is oxidised to nitrite (${\mathrm{NO}}_{2}^{-}$) by AOB and AOA activity. In the second stage, nitrite is oxidised to nitrate (${\mathrm{NO}}_{3}^{-}$) by NOB activity [6,7,9,12,13]. As a result, nitrite and nitrate concentrations in water increase. Recent studies suggest that some microbes via a single stage can completely oxidise ammonia to nitrate without forming a nitrite intermediate [14,15]. Once the nitrification process initiates and no preventive actions are taken, it becomes severe, which is difficult to control [2,16,17,18].

Uncontrolled nitrification can disrupt the biological stability of the distribution system, which may cause several water quality issues [19,20]. For example, increased bacterial activity during nitrification can drop the system pH, which can promote corrosion of distribution system materials [19,20,21]. Nitrifying bacteria promote the growth of heterotrophic biofilms that adversely affect the taste and odour of water by producing metabolic by-products [22]. On the other hand, incomplete nitrification of ammonia can increase the toxic nitrite level in water. It has been found that the nitrifying bacteria and other heterotrophic bacteria can release soluble microbial products (SMPs), which are organic in nature, produced through substrate metabolism (associated with biomass growth and biomass decay) [23,24,25,26]. These SMPs can further accelerate the chloramine decomposition, causing a rapid loss of chloramine residual within a short time [23,25]. Nitrifying bacteria and other heterotrophic bacteria may also release extracellular polymeric substances (EPS), which are a kind of matrix embedded within the biofilm. An EPS layer can provide some level of protection to the bacterial cells against the disinfectant and the outer environment, hence, microbes become more resistant to disinfectants [27,28,29].

Water utilities adopt routine monitoring plans to assess the water quality and the likelihood of nitrification [12,30]. Biomonitoring has the potential to detect the onset of nitrification at an earlier stage, before sufficient levels of chemical surrogates are accumulated in the system [30]. The biomonitoring process requires conducting various microbiological tests such as most probable number (MPN) for heterotrophic nitrifying bacteria, cell mass counting, fluorescent antibody tests, next-generation sequencing (NGS), flow cytometry, fluorescence in situ hybridisation (FISH), and polymerase chain reaction (PCR) [10,12,31,32,33,34,35,36,37,38,39]. However, some of these microbiological tests are culture-based methods that require a lengthy incubation period to obtain a reliable result [12,40]. For example, the MPN method requires one to incubate the diluted samples for at least 3 to 15 weeks. Alternatively, microbiological activity can be monitored by assessing various surrogate parameters that affect the growth of nitrifiers [1,7,9,10,41]. Some of these parameters include pH, free ammonia, monochloramine or total chlorine residual concentration, alkalinity, dissolved oxygen (DO), nitrate, and nitrite [1,2,9]. During the various stages of nitrification, these parameters are observed to change, which indicate the changes in the microbiological activity. For instance, ammonia oxidation by AOB, AOA, and NOB produce acids which may drop the system pH [9]. Similarly, nitrifying bacteria consume oxygen which reduces the DO level in water, or an elevated level of nitrite and nitrate is accumulated into water as a result of ammonia oxidation by nitrifying bacteria [1,7,9]. Monitoring surrogate parameters may not be able to detect the onset of nitrification at an earlier stage. Apart from these methods, water utilities use microbial decay factor, which is defined as the ratio of microbiological decay coefficient to the chemical decay coefficient [6,42,43]. The value of the microbial decay factor is often site-specific, and usually less than one, meaning there is partial or no nitrification in the system. However, a value greater than one does not mean that nitrification occurred definitely; rather, it indicates that the system is vulnerable to rapid disinfectant decay due to enhanced microbiological activity [6,25,42,43]. In contrast, Hossain, et al. [13] recently developed a spectrophotometry-based method to assess nitrification in bulk samples, which can be extended to monitor the same in a WDS. All these available methods have advantages and disadvantages; hence, they need to be considered in context for the specific WDS when implementing a nitrification monitoring plan.

The intensity and propagation of nitrification in a WDS can be prevented by maintaining an adequate level of disinfectant residual [7,9,30]. However, once nitrification starts, chloramine is rapidly decomposed within a short time. Current operational practices that have been adopted to control nitrification include reducing water age, raising the pH of water, increasing the chlorine-to-ammonia ratio, flushing the distribution system regularly, optimisation of natural organic matter (NOM) removal during water treatment process and applying breakpoint chlorination at regular intervals [7,17,44,45,46]. It has been found that nitrifiers’ growth is inhibited by element toxicity. Metals such as zinc (Zn), copper (Cu), and silver (Ag) can inhibit nitrification to some extent [47,48,49,50,51,52]. On the other hand, the application of low levels of phosphate (${\mathrm{PO}}_{4}^{3-}$) (usually less than 5 μg $L^{-1}$) is found to inhibit nitrification [53]. Similarly, in an activated sludge system, the use of graphite nanoparticles (GNPs) slows down the nitrification process [54]. Moreover, some studies suggest that using chlorite (${\mathrm{ClO}}_{2}^{-}$) at a concentration of 0.8 mg $L^{-1}$ can effectively inhibit the growth of nitrifying bacteria [55,56]. While some of these strategies have been promising in controlling nitrification, the performance is often site-specific, as the treated water characteristics from water treatment plants (WTPs) vary [56]. For example, a long-term use of breakpoint chlorination may increase the chlorinated and nitrogenous DBPs’ levels in such distribution systems where NOM concentration is relatively high [57,58]. Similarly, the application of phosphate as a control measure of nitrification can reduce the copper and zinc levels, which increases the likelihood of nitrification [53]. Hence, for efficient control of nitrification, different approaches should be investigated to determine the best practice for each WDS.

This paper aimed to identify gaps in the current literature. A few reviews exist on different aspects of nitrification in drinking water, wastewater, and premise plumbing systems [12,59,60,61]. This study particularly focused on nitrification monitoring and control in drinking water systems. A review in this specific area would be beneficial to chloraminated drinking water systems. We have critically reviewed the current understanding of nitrification process, available monitoring and control strategies, and advantages and disadvantages by these methods to improve the current practices.

## 2. Current Understanding of Nitrification

Drinking water treatment processes that involve chloramine disinfectants often experience nitrification. Chloramines have three different chemical forms: monochloramine (${\mathrm{NH}}_{2}\mathrm{Cl}$), dichloramine (${\mathrm{NHCl}}_{2}$), and trichloramine or nitrogen trichloride (${\mathrm{NCl}}_{3}$) [3,62]. Operating conditions at the treatment plant are controlled such that monochloramine becomes the dominant forming species. According to a survey in USA, 63% utilities that are operated with monochloramine experience nitrification, typically more frequently in the summer season [9]. Microbiological monochloramine decay can be related to nitrification involving AOB, AOA, NOB, and possibly other unknown bacterial activities. While monochloramine is transported through the water system, it is subjected to decay due to auto-decomposition and chemical reactions with NOM and other species. This way, free ammonia is released into the distribution system [63]. Free ammonia can also enter the distribution system during the chloramination process if an optimum mixing ratio of chlorine and ammonia is not maintained. The following set of reactions (Table 1) shows the major pathways for ammonia release in a typical drinking water distribution system (DWDS) [64,65].

In the first step of the biological oxidation process, this free ammonia is oxidised to nitrite by AOB, AOA, and in the second step, nitrite is oxidised to nitrate by NOB [9,12,66,67]. The approximate reactions involved in the nitrification process is given below.

(1) $$\begin{array}{c} \begin{array}{c} 2{\mathrm{NH}}_{3}+3O_{2}\overset{\left(\mathrm{AOB},\;\mathrm{AOA}\right)}{\to }\;\;2{\mathrm{NO}}_{2}^{-}+2H_{2}O+2H^{+}\\ \;\;↓ \end{array}\\ 2{\mathrm{NH}}_{4}^{+}+3O_{2}\overset{\left(\mathrm{AOB},\;\mathrm{AOA}\right)}{\to }\;\;2{\mathrm{NO}}_{2}^{-}+2H_{2}O+4H^{+}\\ \begin{array}{c} \;\;↓\\ \;\;\;\;+{\;O}_{2}\overset{\left(\mathrm{NOB}\right)}{\to }2{\mathrm{NO}}_{3}^{-} \end{array} \end{array}$$

Typical drinking water systems can experience both complete and incomplete nitrification. A complete nitrification results in the consumption of alkalinity (${\mathrm{HCO}}_{3}^{-}$), formation of carbonic acid ($H_{2}{\mathrm{CO}}_{3}$), and increased biomass ($C_{5}H_{7}O_{2}N$) and nitrate concentration [68]. The overall reaction involved in complete nitrification process is given below.

(2) $${\mathrm{NH}}_{4}^{+}+3.3{\;O}_{2}+6.708{\;\mathrm{HCO}}_{3}^{-}\to 0.129{\;C}_{5}H_{7}O_{2}N+3.373{\;\mathrm{NO}}_{3}^{-}+1.041{\;H}_{2}O+6.463{\;H}_{2}{\mathrm{CO}}_{3}$$

**Figure 1:**
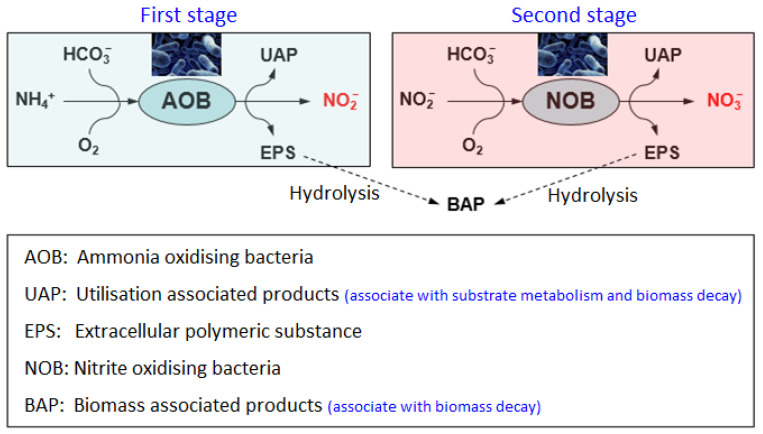
Microbiological process involved in nitrification (based in part on Ni et al. [24], Copyright 2011, Elsevier).

If incomplete nitrification occurs, nitrite can accumulate in the system which is chemically reactive to monochloramine and accelerates its decomposition process, particularly in the presence of bromide [63,69]. Consequently, more free ammonia is released into the distribution system. This increased free ammonia accelerates the growth of microbes, which accelerates the production of nitrite and, ultimately, the rate of monochloramine decay is increased and the whole process is repeated. Once the nitrification process starts, it can lead to a rapid or complete loss of monochloramine residual from levels greater than 2 mg $L^{-1}$ to below the detection limit within a few weeks [10,16]. Nitrification is found to slow or cease at temperatures below 10 °C or above 40 °C. The most favourable temperature for nitrification is between 25 to 30 °C [8]. The whole nitrification process is illustrated in Figure 1.

AOB that oxidise ammonia to nitrite include *Nitrosomonas*, *Nitrosococcus*, and *Nitrosospira*. Two key enzymes, (i) ammonia monooxygenase (AMO) and (ii) hydroxylamine oxidoreductase (HAO), are believed to drive the ammonia oxidation to nitrite. The first enzyme is an integral membrane type protein that oxidises ammonia to an intermediate product, hydroxylamine, and the second enzyme is a periplasmic type that converts hydroxylamine to nitrite [59]. These two enzymes act sequentially so that the cell can successfully complete the oxidation process and generate energy.

Similarly to AOB, there is a particular enzyme named nitrite oxidoreductase (NXR), which is an integral membrane protein allowing NOB to perform the oxidation of nitrite to nitrate [59]. The structure of NXR enzyme is composed of three subunits, nxrA, nxrB, and nxrC. Depending on the NOB species, the nxrA and nxrB subunits can be either periplasmic or cytoplasmic enzymes, while the nxrC spans the cytoplasmic membrane [70]. The difference in the enzyme structure is possibly one of the major causes for NOB being such a diverse group of bacteria, which includes *Nitrobacter*, *Nitrospina*, *Nitrococcus*, and *Nitrospira* [59]. Among them, the *Nitrospira* spp. and *Nitrobacter* spp. are largely found in most drinking water systems [67,71]. The structural difference in the NXR enzyme, being either periplasmic or cytoplasmic, may also result in the differences in environmental adaptation for NOB [59]. The periplasmic NOB can survive in low nitrite environments, while the cytoplasmic NOB requires high nitrite levels [70].

Many studies have reported the presence of high densities of heterotrophic bacteria, ranging from $10^{3}$ to $10^{5}$ colony forming units (CFU) per millilitre in drinking water samples [72,73]. The disinfection strength of water largely dominates the abundance and distribution of nitrifying bacteria in most DWDS. A study by Cunliffe [16] reported to find nitrifying bacteria in 64% samples collected from several water utilities in South Australia. The study showed that when the monochloramine residual concentration was low (≤1.0 mg $L^{-1}$), nitrifying bacteria were found more often (83% samples), whereas when the monochloramine concentration was high (>5 mg $L^{-1}$), nitrifying bacteria were present in 20.7% of samples. Wolfe et al. [10] found higher concentrations of AOB in reservoir and pipe sediments when compared to pipe biofilm. Similarly, Lipponen et al. [74] studied the occurrence of nitrifying bacteria in several WDS of different origins and treatment practices and found high number of nitrifying bacteria in pipe sediment. The AOB species dominates in parts of the WDS with low water age and sufficient monochloramine residuals, whereas the NOB species mainly dominates in areas with higher water age and depleted monochloramine levels [75].

Under suitable conditions, typical nitrifiers grow slowly, having generation time from 8 h to several days [76]. The growth of nitrifiers depends on several factors including pH, temperature, light, substrate concentration (ammonia for AOB), and DO. Nitrifiers’ growths are favourable at temperatures between 15 to 30 °C and a pH between 6.5 to 10 [10]. They can increase their growth rate and cell mass by incorporating organic compounds such as glucose, acetate, formate, and yeast extract [76]. Nitrifiers can discharge organic compounds that enhance heterotrophic bacterial growth [76]. During the stage of severe nitrification, a rapid or complete loss of monochloramine residual is observed, suggesting that there might be some other mechanism involved other than nitrifying bacteria. To explain this phenomenon, researchers including Krishna and Sathasivan [66], Bal Krishna, et al. [23], and Sawade, et al. [25] suggested that nitrifying bacteria together with other heterotrophic bacteria may produce SMPs that boost the chloramine decay process. The SMPs are organic in nature that are released during the interaction at various stages with the environment, including substrate metabolism, bacterial growth, bacterial lysis, and degradation [77]. SMPs are broadly classified into two categories: (i) utilisation-associated products (UAP), which are associated with substrate metabolism and biomass growth, and (ii) biomass-associated products (BAP), which result from biomass decay [26]. In addition, nitrifying bacteria may also release EPS, which is a key component of biofilm, providing structural and functional integrity to the biofilms. An EPS layer surrounding the bacterial cells can serve as a barrier and provide a certain degree of protection to the bacterial cell from environmental stresses such as UV radiation, pH shifts, freezing, heat, osmotic shock, predator attack, and disinfectants.

**Figure 2:**
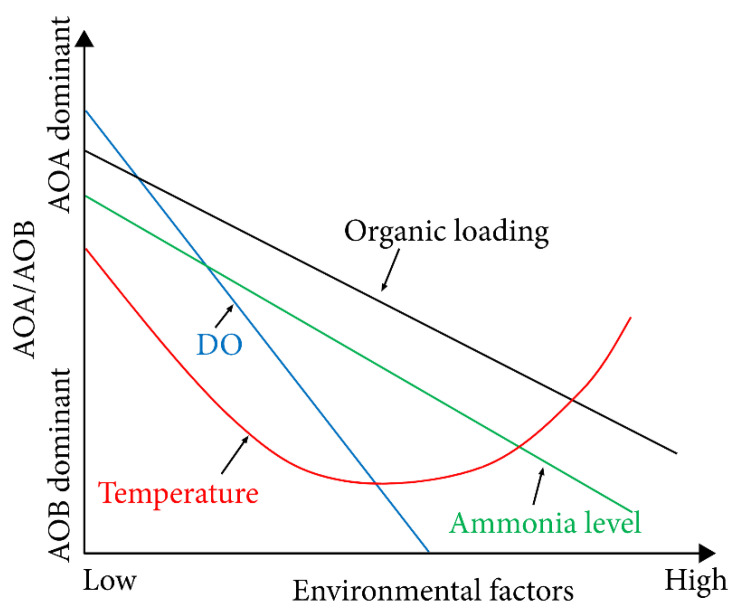
AOA/AOB distribution for varying environmental factors (ammonia, organic loading, oxygen level, and temperature) (based on [11,82]).

Previously, it was believed that the ammonia oxidation process is mainly governed by AOB activity [9,67]. However, in recent years, the *amoA* gene driving the ammonia oxidation process has been identified in large numbers of archaea distributed in the marine environment, indicating that they also have the capability to oxidise ammonia to nitrite [78]. These archaea are commonly referred to as AOA, which are mainly found in wastewater systems. Roy, et al. [79] observed that the abundance of the archaeal *amoA* gene in the biofilm samples of municipal wastewater is two to three orders of magnitude higher than bacterial *amoA* gene. A similar study by Chen, et al. [80] confirmed the abundance of AOA over AOB in a municipal wastewater system. AOAs in most DWDS are closely related to the *Nitrosopumilus maritimus* species, in which cell dimensions are 10 to 100 times smaller than typical AOB cells. Thus, the ammonia oxidation by AOA could be 10-fold lower than that of AOB [81]. As shown in Figure 2, Yin, et al. [11] and Guo et al. [82] hypothesised the abundance of AOA and AOB in water, based on several environmental factors including temperature, organic loading, ammonia, and oxygen levels. AOA are highly adapted to energy-stressed environments and can survive with very low ammonia levels, far below than those required for AOB survival [83]. Thus, ammonia oxidation by AOA can dominate over AOB in systems with low nutrient levels.

In drinking water systems, biofilm can gradually develop in pipes through a multistage process, including cell attachment onto a wall’s surface, production and exertion of EPS material, colonising and development of biofilm community, cell maturation, and finally, detachment. The exact nature of EPS composition greatly varies depending on microbial strain. However, within a few days, the cells can colonise and develop their community [84]. The biofilm density in DWDS continuously changes due to the changing hydraulic condition and nutrient levels [85]. Biofilm growth is found to be increased with increased nutrient levels under turbulent flow conditions when compared to laminar flow with low nutrient levels [86]. The mature biofilm finally reaches a quasi-steady state, where the growth is balanced by various losses, such as detachment due to mechanical stress, release by predator attack, loss through erosion, and self-induced dispersal from starved subregions [84]. The detachment allows bacteria to attach and form a biofilm onto a new surface, or to join another biofilm community downstream of the original community. Biofilms have the potential to harbour a high density of bacteria, ranging from $10^{4}$ to $10^{7}$ CFU ${\mathrm{cm}}^{-2}$ [87]. Heterotrophs and nitrifiers are the most dominant microbes living in biofilms. These biofilm bacteria, including AOB and NOB, can promote rapid microbiological decay and nitrification in drinking water systems [9,12,67].

The EPS matrix can act as an effective barrier, protecting microbes living inside the biofilm. However, research suggests that monochloramine is highly effective in penetrating biofilms. In fact, monochloramine is shown to be 2 to 100 times less effective, while free chlorine is up to 3000 times less effective in inactivating biofilm bacteria when compared to the same inactivation in bulk water [88]. Both ammonia and nitrite oxidation are aerobic reactions that require the presence of oxygen to proceed. The DO content in water is the primary source of oxygen to perform these aerobic reactions. In the absence or at low concentration of monochloramine, the biofilm’s matrix can prevent or allow very little amounts of oxygen to reach the internal portions of the biofilms, and hence the majority of the aerobic activity occurs at the surface of the biofilm [89]. Once monochloramine residual concentration increases, the DO can reach deep inside the biofilm’s matrix via monochloramine penetration, and aerobic activity starts promoting nitrification. In addition to aerobic microbes, biofilms in DWDS can possibly harbour a significant portion of anaerobic bacteria, including anaerobic ammonia-oxidising (anammox) bacteria [90]. This bacterium is capable of oxidising ammonia to nitrite and nitrate under anoxic or low oxygen conditions. The anammox bacteria are most likely to be seen in a disinfectant-free system or under severe nitrifying conditions, where the aerobic activity is limited at the biofilm surface and ceases at the inside portions of the biofilm because of inadequate oxygen supply. Currently, most anammox isolates have been associated with the wastewater treatment process, however, there is no firm evidence of anammox activity in DWDS.

While in the last few decades, nitrification was believed to be a two-step microbiological process performed by AOB and NOB, recent studies by Daims, et al. [14] and Van Kessel, et al. [15] found that novel *Nitrospira* species are fully capable of oxidising ammonia to nitrate without a nitrite intermediate. These species are named as COMplete AMMonia OXidisers (comammox), which have been identified in many ecosystems including natural waterbodies and terrestrial ecosystems. These comammox cells contain specific genes including *nxr*, *amo,* and *hao* genes that are required to perform the complete ammonia oxidation to nitrate. However, not all *Nitrospira* species have the capability of complete ammonia oxidation [14]. Under normal conditions, the comammox are difficult to detect using traditional methods, e.g., amoA qPCR primers, because the AMO enzymes in comammox are genetically arranged in a different pattern than usual AOB AMO enzymes. Comammox are slow-growing microorganisms, having a low rate of ammonia oxidation and high yield. The distribution and abundance of comammox in drinking water systems are currently unknown. However, evidence suggests comammox in drinking water systems, but no definitive proof has been found yet [59,91].

## 3. Monitoring Rapid Chloramine Decay and Nitrification

During nitrification, enhanced microbiological activity in water can be assessed in several ways such as conducting microbiological tests or assessing surrogate parameters or assessing the microbial decay factor or the application of a nitrification model. These are discussed below.

### 3.1. Assessing Microbiologically Mediated Chloramine Decay Using Microbiological Tests

Most bacteria in drinking water are a few micrometres in cell length, with a wide variety of shapes characterised as spheres, rods, and spirals. Their cell composition varies by the cell wall, cell membrane, ribosomes, and nucleoid (DNA). They can be classified based on cell structure, cellular metabolism, or the differences in cell components such as DNA, fatty acids, pigments, antigens, and quinones [12]. Some of the existing microbiological tests require culturing bacteria for several weeks. Because of the limitations of culture-based methods, nucleic acids components have been increasingly used to detect and identify bacteria [92]. Nitrifiers can be identified by targeting the 16S rRNA (or 16S rDNA) gene. The DNA regions with highly variable nucleotide sequences are usually targeted. In addition, the AOB, which contain the specific *amoA* gene, are increasingly used for identification and characterisation [33,92,93]. Below are some commonly used biological techniques to characterise and quantify most nitrifying bacteria in drinking water.

#### 3.1.1. Most Probable Number (MPN)

The most probable number (MPN) is a statistical method used to estimate the concentration of viable bacteria in a sample by replicating the liquid broth growth in ten-fold dilutions. The MPN method is based on several assumptions: (i) the bacteria are randomly distributed over the sample; (ii) the samples in different tubes are independent; (iii) the bacteria are not clustered together but exist and are spread individually over the sample; (iv) bacteria in every tube of sample should produce a detectable growth or change in concentration level. Nitrifying bacteria, including ‘*Nitrosomonas europaea*’, ‘*Nitrosospira*’, and ‘*Nitrobacter*’, are frequently classified by this technique using a media selective for ammonia or nitrite oxidisers. Usually, the medium for AOB cultivation is prepared using ${\left({\mathrm{NH}}_{4}\right)}_{2}{\mathrm{SO}}_{4},{\;\mathrm{NH}}_{4}\mathrm{Cl},{\;\mathrm{KH}}_{2}{\mathrm{PO}}_{4},\;\mathrm{KCl},{\;\mathrm{MgSO}}_{4}\times 7{\;H}_{2}O,{\;\mathrm{CaCl}}_{2}\times 2{\;H}_{2}O,\;\mathrm{NaCl}$, and trace element solution, while the same for NOB cultivation are prepared from ${\mathrm{NaNO}}_{2},\;\mathrm{NaCl},{\;\mathrm{MgSO}}_{4}\times 7{\;H}_{2}O,{\;\mathrm{KH}}_{2}{\mathrm{PO}}_{4},{\;\mathrm{CaCO}}_{3}$, with a trace element solution [94,95,96,97]. The diluted samples are incubated for at least three weeks for ammonia oxidisers and up to 15 weeks for nitrite oxidisers. The MPN technique is reported to have low recovery efficiencies, ranging from 0.1 to 5% [40]. Another major concern of the MPN method is that it requires many replicates with appropriate dilution level to obtain a narrow band of confidence interval, and the method requires a lengthy incubation period. Despite these limitations, the MPN method is widely adopted to classify a range of bacteria in water samples, including nitrifying bacteria [10,98].

#### 3.1.2. Fluorescence in Situ Hybridisation (FISH)

Fluorescence in situ hybridisation (FISH) is a molecular technique to identify and visualise specific bacteria in both natural and engineered environments. This method targets particular sequences of nucleic acids (16S rRNA) and involves the fixation of cells to microscope slides, hybridisation of fluorescently labelled probes to complementary target sequences, and analysis of fluorescence signals [33,92,93]. However, this method is inefficient to process many samples in parallel, because the bacterial cells are usually fixed and hybridised on microscope slides instead of processing them in solutions. In addition, cell fixation and hybridisation could be challenging for specific environmental samples and Gram-positive cells. Some studies suggest that FISH is inadequate to detect AOB activity using 16S rRNA, because, for many AOB, the rRNA and mRNA component under low nutrient conditions was not found to be lower than nutrient-rich conditions [33,93]. Also, false positives and false negatives may result from the lack of specificity and low fluorescent responses [99].

#### 3.1.3. Next-Generation Sequencing (NGS)

Next-generation sequencing (NGS) allows massively parallel sequencing of nucleotides in an entire genome or target regions of DNA or RNA. This method has been extensively used for the investigation of environmental microbiomes [38,39]. Application of NGS in drinking water systems can provide insight into microbial communities and their functional capacities, and potentially helps to monitor bacterial growth and contamination in the system [35,39]. A typical NGS process involves four major steps: (i) library preparation; (ii) amplification; (iii) sequencing; (iv) data analysis. In the first step, the target DNA is extracted from the sample and a library is created by fragmenting DNA, and by adding specialised adapters to both ends. These adapters contain complementary sequences, allowing the DNA fragments to bind to the flow cell. In the second step, the library is amplified using clonal amplification methods and PCR to increase the signal. In the third step, all DNA in the library is sequenced using a sequencing instrument. It generates a huge amount of data which are interpreted using the instrument software. A major advantage of NGS method is its higher sensitivity to detect low-frequency variants. However, the NGS method yields massive amount of data that require expert analysis to produce a concise result.

#### 3.1.4. Flow Cytometry Method (FCM)

Flow cytometry is a rapid, reproducible, and robust method for determining bacterial concentrations in drinking water [34,37]. It uses a laser light source to produce optical signals, scattered from particles, or emitted from auto-fluorescing cells or fluorescently labelled cells, for their identification and quantitative measurement. In this process, the sample containing cells or particles is suspended in a sheath fluid and allowed to pass in a single file through the centre of the focused laser beam to measure its optical properties. The detector measures forward-scattered light, side-scattered light, and dye-specific fluorescence signals, which are then converted into electronic signals and processed by a computer to gather information. A variety of fluorescent reagents are used in flow cytometry. Some of these reagents include viability dyes, DNA binding dyes, nucleic acid dyes, fluorescently conjugated antibodies, and fluorescent proteins [36]. The sheath fluid commonly used are phosphate-buffered saline, hepes-buffered saline, and 0.1% of 2-phenoxyethanol that help to reduce surface tension. It has been found that FCM-based total bacterial cell counts in drinking water samples correlate well with hydraulic retention time [37]. A major advantage of FCM is that it has the potential to characterise individual cells in a population without their averaging. An FCM instrument with fluorescence-activated cell sorting capabilities allows one to recollect the cells of interest that have run through the flow cytometer for further analysis. However, the FCM instrument can be very sophisticated and prone to microfluidics system blockages. It does not work well for cells that tend to stick together.

#### 3.1.5. Polymerase Chain Reaction (PCR)

Nitrifiers can also be identified and quantified by using polymerase chain reaction (PCR), targeting the 16S rRNA genes (16S rDNA) and *amoA* gene sequences [31,32]. The target region of the DNA is amplified up to a factor by $10^{9}$ with a high level of accuracy [100]. The major steps in PCR involve the extraction of target DNA, amplification, and sequencing [31,32]. A major concern of the PCR technique is that it detects nucleic acid components and does not consider whether they come from viable cells. Contamination of samples by humic substances and metals can also lead to a false-positive results in PCR analysis. Other molecular techniques such as terminal restriction fragment length polymorphism (T-RFLP) and denaturing gradient gel electrophoresis (DGGE) can also be used to determine the diversity of nitrifiers [67].

#### 3.1.6. Fluorescent Antibody Test

The fluorescent antibody (FA) technique is useful to visualise and identify certain groups of bacteria in environmental samples that are difficult to isolate or culture. Nitrifier strains are detected by identifying the presence of a particular antigen (typically a specific protein on the surface of bacteria). Different nitrifier strains require different FAs to detect them [46]. This method involves attaching fluorescent chemicals (fluorophore) to the constant region of an antibody to visualise the target distribution under a fluorescent microscope. The FA method is classified into the direct fluorescent antibody (DFA) and the indirect fluorescent antibody (IFA). The DFA method uses a single antibody directly conjugated to a fluorophore to stain the target protein. The IFA test uses two antibodies, where the primary antibody is used to bind the target protein, which is then detected using a conjugated secondary antibody. A limitation of this method is that FAs can have non-specific binding to EPS [101].

#### 3.1.7. Cell Mass Counting

Nitrifiers’ concentrations can also be determined by directly counting the cell numbers under a microscope using a hemocytometer or a counting chamber [32,102]. A hemocytometer consists of a microscope slide with many gridlines of specified dimensions so that the intermediate area between the lines are known. The bacterial concentration is calculated by counting the number of cells per unit area of the grid and multiplying it by a conversion factor that depends on the chamber volume and the dilution factor. The direct cell count method, however, has many limitations, such as it only counts the total cell numbers and does not distinguish between live cells and dead cells, and that it may fail to detect small cells that are not visible under the microscope. Nitrifiers or a specific group of bacteria are also difficult to distinguish in a mixed culture. This method is suitable when the abundance of nitrifying bacteria dominates over other bacteria in the sample. Optical density or turbidity can also be used to measure the biomass concentration. Groeneweg, et al. [103] found a linear relationship between optical density and dry matter concentration. However, during nitrification, turbidity was found to be increased in some cases, whereas it remained same in most cases [9].

### 3.2. Assessing Rapid Chloramine Decay and Nitrification Using Surrogate Parameters

**Figure 3:**
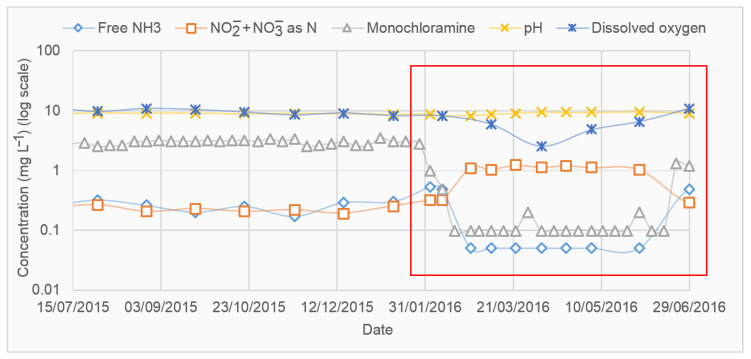
Water quality changes during a nitrification episode in a DWDS in South Australia. The plot represents grab samples test data at regular intervals. The left side of the plot indicates usual water quality and no nitrification, while the data within the red rectangle shows a sudden change in water quality during the nitrification event. Monochloramine, free ammonia, and DO levels dropped, while nitrite and nitrate levels increased. The water quality at the end of the plot returned to normal after preventive measures were taken.

Nitrification may degrade the water quality of the distribution system [6,12,46]. It can directly impact water quality in many ways, such as by consuming DO, decreasing pH, or increasing the nitrite and nitrate proportion in water. The indirect impacts include increasing heterotrophic bacterial growth, loss of monochloramine residual, or increasing the formation of DBPs while flushing the distribution system using free chlorine as a nitrification control strategy. Figure 3 shows the water quality changes during a nitrification event recorded in a DWDS located in South Australia. It is evident in Figure 3 that nitrification causes a sudden drop of monochloramine, free ammonia, and DO concentrations, while nitrite and nitrate concentrations are increased. Nitrification is most likely to occur in specific areas of the distribution system of low water velocity, such as tanks and dead-end mains. Monitoring of water quality parameters in these locations can help to detect nitrification at an earlier stage. Since microbiological monitoring of nitrifying bacteria is complicated, inefficient, and time-consuming, water utilities use surrogate parameters such as free ammonia, nitrate, nitrite, monochloramine residual concentration and heterotrophic plate count (HPC). There is no such single surrogate parameter that exists that perfectly indicates the nitrification; rather, multiple parameters need to be evaluated to ensure the nitrification status. A list of some of these surrogate parameters, as mentioned by Wilczak, et al. [9], is presented in Table 2.

Water utilities monitor a combination of these parameters based on their individual system experience. According to Kirmeyer, et al. [7], the best way to monitor nitrification is to monitor the chemical balance of nitrogen species such as free and total ammonia, nitrite, and nitrate in the distribution system. The nitrogen balance is checked by using the following formula.

(3) $$\mathrm{Nitrogen}\;\mathrm{balance}={\mathrm{Free}\;\mathrm{NH}}_{3}+{\mathrm{NO}}_{2}^{-}\;\mathrm{as}\;N+{\mathrm{NO}}_{3}^{-}\;\mathrm{as}\;N+0.27{\;\mathrm{NH}}_{2}\mathrm{Cl}$$

Under normal operating conditions, this number can fluctuate. However, if increased nitrite and nitrate levels and an increased nitrogen balance are observed, it is likely that a nitrification episode will happen.

Scott, et al. [105] assessed several water quality parameters including pH, temperature, organic carbon, total chlorine, and ammonia, and found a positive correlation between nitrifiers and ammonia levels, while finding a negative correlation between total chlorine and nitrifiers and HPC. They reported a strong correlation between HPC and AOB and suggested HPC to be an operational parameter to assess the microbiological condition in the distribution system. Shi, et al. [28] assessed several nitrification indicators and suggested that the changes of nitrite, total organic carbon (TOC), and turbidity levels can efficiently indicate nitrification potential. A critical threshold residual (CTR) was defined by Pintar, et al. [106] as a more general indicator of nitrification. When the nitrite, as N accumulation, exceeds 0.05 mg $L^{-1}$, it is likely that a nitrification episode will happen [7,40]. However, this limit is often site-specific, and 0.05 mg $L^{-1}$ ${\mathrm{NO}}_{2}^{-}\;\mathrm{as}\;N$ is too high to be used as an indicator of nitrification. Sathasivan, et al. [18] suggested that the CTR should be identified through bench-scale chloramine decay studies. CTR is the point in the curve where the decay coefficient starts increasing rapidly due to increased bacterial activity. Bradley, et al. [59] mentioned that the CTR is likely to appear within the range of 0.20 and 0.65 mg $L^{-1}$ of total chlorine residual. Scott, et al. [107] suggested that the CTR should appear at 0.50 mg $L^{-1}$ of total chlorine residual. Apart from these methods, currently, many water utilities use microbiological decay factor ($F_{m}$) to be an indicator of rapid monochloramine decay and nitrification [6,25,42,43].

### 3.3. Monitoring Microbiological Decay Factor (F_m_)

The chemical and microbiological component of chloramine decay is difficult to separate, because it requires quantifying and measuring complex chemical and microbiological properties. While water utilities use surrogate parameters for the rapid assessment of microbiologically mediated chloramine decay, these parameters do not directly quantify the part of chloramine decay caused by microbiological activity. Knowing these limitations, Sathasivan, et al. [6] developed an innovative method to distinguish the chemically and microbiologically mediated chloramine decay. The proposed method provides a more quantitative result than traditional methods [43]. In this method, two water samples are prepared: unprocessed and processed. The unprocessed sample is the original water obtained from the WTP, while the processed sample is obtained by filtering the unprocessed sample through a 0.2 µm filter paper, or is microbiologically inhibited by adding 100 μg of Ag $L^{-1}$ as silver nitrate. It is reported that most nitrifying bacteria in treated water are rod-shaped cells with width ranging between 0.3 to 0.6 μm and length between 0.8 to 3.0 μm [10,108]. Therefore, it is expected that 0.2 µm filtration will retain those bacteria and theoretically produce a water sample that will be free from nitrifying bacteria. Monochloramine is then added to both samples and its decay is observed. The decay in the unprocessed sample is due to both chemical and microbiological activities, whereas decay in the processed sample is subjected to chemical reactions only. If the decay in the unprocessed and processed samples are $k_{t}$ and $k_{c}$, respectively, then,

$$\mathrm{Total}\;\mathrm{decay}\;(k_{t})=\mathrm{microbiological}\;\mathrm{decay}\;(k_{m})+\mathrm{chemical}\;\mathrm{decay}\;(k_{c})$$

$$\mathrm{Therefore},\;\mathrm{microbiological}\;\mathrm{decay},\;k_{m}=k_{t}-k_{c}$$

The term $k_{m}$ and $k_{c}$ are referred to as microbiological and chemical decay coefficients, respectively, which can be determined through data analysis and curve fitting. Chemical decay mainly includes chloramine auto-decomposition and decay due to the reaction with NOM. Instead of quantifying microbiologically mediated chloramine decay, Sathasivan, et al. [6] introduced a new term to provide a point of reference, called microbial decay factor ($F_{m}$).

(4) $$F_{m}=\frac{k_{m}}{k_{c}}$$

$F_{m}$ can be interpreted as the ratio of the rate of chloramine decay due to microbiological activity to the rate of chemical decay at any time in the unfiltered sample. Usually, chloramine decay after 7 days is considered to determine the value of $F_{m}$ [42]. The theoretical minimum value of $F_{m}$ is zero, which indicates no microbiologically mediated chloramine decay, whereas it increases due to increased impact of microorganisms. Currently, the microbial decay factor is increasingly used to monitor nitrification status in WDS [25,42,109]. Regular monitoring of $F_{m}$ may provide an early warning for the onset of nitrification and utilities can take necessary measures to lower the $F_{m}$ value [25,42,109]. During the various stages of nitrification, the value of $F_{m}$ is reported to change, as presented in Table 3.

A high value of $F_{m}$ does not mean that nitrification occurred; rather, it indicates the system is susceptible to rapid monochloramine residual loss due to microbiological activity [25].

### 3.4. Other Approaches to Assess Nitrification

Fleming, et al. [98] observed that total chlorine residual and free ammonia concentrations can affect the microbial growth and inactivation kinetics in a WDS, and the right combination of these factors can lead to a nitrification episode. They developed a nitrification potential curve where Monod kinetics were used to model the AOB growth, and Chick–Watson law was used to model the AOB inactivation by chloramine. The application of the nitrification potential curve to a full-scale drinking water system suggested that the maximum chloramine residual at which a nitrification episode may start ranges between 1.6 to 2 mg $L^{-1}$, depending on pH value. Similarly, Speitel, et al. [110] developed a nitrification index (NI), which is defined as the ratio of AOB growth rate to their inactivation rate by chloramine. A value of NI greater than one means nitrifiers’ growth rate dominates over their inactivation rate. Hence, monitoring the NI value regularly may provide an early warning of nitrification and utilities can take necessary measures to lower the NI value. Alternatively, Moradi, et al. [111] proposed a chloramine decay index (CDI), which is defined as the ratio of UV light absorbance at 230 nm to that at 254 nm. During nitrification, the UV absorbance at 230 nm will increase due to the production of nitrate, nitrite, and SMPs, while the same will decrease at 254 nm, due to organic consumption by nitrifying bacteria. A CDI value greater than one indicates more microbiological decay or the likelihood of a nitrification episode.

Gomez-Alvarez and Revetta [30] assessed the feasibility of microbiome-based bioindicators to monitor nitrification in DWDS. Bioindicators are the biological features of living organisms in water (e.g., species, population, community) that can be used to indicate the quality and stability of their ecosystem. Bioindicators were identified from high-resolution taxonomic profiles generated using 16S rRNA gene dataset. The analysis of microbiomes indicated a variety of bacterial populations in nitrified samples. A supervised machine learning model trained with bioindicator profiles was used to classify test samples subjected to nitrification. Data analysis suggested that using a combination of different bioindicators was able to classify 85% of nitrification instances. A major advantage of this bioindicator-based monitoring is that it can provide early detection of nitrification when compared to traditional monitoring, where sufficient levels of chemical surrogates are required to detect nitrification. The bioindicator-based monitoring assesses the microbial populations responsible for producing chemical surrogates. Currently, the proposed method requires additional validation to confirm its applicability in real drinking water systems.

Yang, et al. [112] developed a nitrification model that simulates a range of chemical and microbiological species, such as nitrifying bacteria, chloramines, ammonia, nitrite, and nitrate, in a pilot-scale system. The model only describes the suspended growth of nitrifiers and does not include a biofilm nitrification component. It incorporates a set of mass balance equations for chloramines, ammonia, active AOB and NOB, nitrite, and nitrate. Model simulation suggested that the microbes responsible for nitrification can be adapted in a low nutrient environment. Currently, the application of the model successfully predicted the onset of nitrification episode in a pilot-scale system, which can be extended to real distribution systems to monitor and manage nitrification. Similarly, Lu, et al. [113] developed a mathematical model to predict the substantial change of several water quality parameters such as oxidised nitrogen, ammonium nitrogen, DO, alkalinity, biomass, and disinfectant residual under laminar and turbulent flow conditions. The model used Monod expression to represent the organic oxidation and nitrification and denitrification, while the Chick-Watson law was used to model the microbial inactivation by chloramine. Application of the model suggested that turbulent flow can lead to more nutrient release from bulk water, which can contribute to more biomass accumulation in pipes. The model can be used to assess the microbiological activity in a WDS.

While transporting through a water network system, microbiological species are continually exchanged between bulk water phase and biofilm. To describe the bacterial growth in bulk water and biofilm, Jegatheesan, et al. [114] developed a mathematical model by assuming major processes, including attachment and detachment of bacteria from biofilm surface, chloramine decay in bulk water phase and in biofilm, and bacterial growth under chloramine inhibition. The limiting factors considered for bacterial growth are substrate availability and disinfectant concentration. The model predicts the organic substrate concentration which can be used to assess the biological stability of water. In contrast, the kinetic model developed by Liu, et al. [115] can be used to model the active AOB and NOB biomass in a WDS. Their model uses Monod expression to represent the AOB and NOB growth, with growth assumed to be limited by chloramine disinfectant and DO availability. The flow in pipes is assumed to be plug flow, while the major source of ammonia for biomass growth is produced through chloramine decomposition. The application of the model was found to adequately predict the ammonia and nitrate concentrations in a pilot distribution system (PDS); however, the nitrite was overestimated. Further refining the model can help to monitor different water quality parameters that indicate nitrification in a WDS.

Nitrification instances are often observed in trickling filters in a WTP. A trickling filter is made up of a bed of crushed rock or other coarse media that mainly operates under aerobic conditions. When the water passes through the pores of the filter, organics are degraded through aerobic oxidation by the biofilm surrounding the filter media. Vayenas, et al. [116] developed a dynamic model that describes the nitrification process in trickling filters. The model assumes that biofilm thickness increases due to bacterial growth and decreases due to bacterial decay. The biomass growth was modelled using Monod kinetics with the ammonia, and nitrite levels were considered as growth-limiting factors. The continuous version of the model was validated by pilot-scale experiments, which suggest that ammonia and nitrite concentrations in the inner matrix of the biofilm are close to zero, while nitrate concentration is maximised. Nitrite can accumulate only when there is a significant ammonia residual observed at the filter outlet. Under both batch and continuous operations, the model can predict the ammonia, nitrite, and nitrate levels along the filter depth as a function of operating parameters, hence, helping to monitor nitrification in filters.

Recently, Hossain, et al. [13] developed a spectrophotometric method to monitor nitrification in a lab-scale system. The method is based on monitoring the changes of relative nitrate and nitrite concentration (combined ${\mathrm{NO}}_{x}^{-}$) in chloraminated water. They observed that in pure monochloramine solutions at various concentrations, the UV absorbance differences at a specific wavelength are related to other wavelengths by a linear function. With the key assumption that spectral absorbance differences of typical chloraminated drinking water will follow a similar function as in chloraminated ultrapure water, the combined ${\mathrm{NO}}_{x}^{-}$ spectra were isolated from the total spectra. Using the support vector regression algorithm (SVR), a model was developed to determine the combined ${\mathrm{NO}}_{x}^{-}$ concentrations from their isolated spectra. To work with this method, the sample is required to keep for a certain period to allow monochloramine decay and ${\mathrm{NO}}_{x}^{-}$ production. After this period, the absorbance difference at various wavelengths due to monochloramine decay is determined based on the absorbance difference at 245 nm using the derived linear function. Finally, the ${\mathrm{NO}}_{x}^{-}$ spectra are isolated using a simple chemometric technique. The method was applied to several test samples, where the nitrified sample showed an increasing trend of ${\mathrm{NO}}_{x}^{-}$ production. The method can be extended to monitor and manage nitrification in drinking water systems.

Another promising method to monitor nitrification is using adenosine triphosphate (ATP), which measures the quantity of active biomass in a water sample. ATP is a coenzyme that drives many processes in living cells. It works with other enzymes to transfer energy to cells by releasing its phosphate groups. ATP production is directly linked with cell growth rate; hence, higher ATP levels indicate greater cell mass [117]. In contrast, cells release their ATP to the outer environment when they become weak and/or lyse, termed as extracellular ATP. The concentration of intracellular ATP is considerably higher than extracellular ATP and could be as much as 1000-fold higher in magnitude [118,119]. The presence of a greater proportion of extracellular ATP indicates a less healthy population. Several methods have been developed to measure the ATP levels, which include colorimetric, fluorescent, and bioluminescent methods, as well as liquid chromatography and mass spectrometry [118,120,121,122,123,124,125]. The bioluminescent ATP assays are widely used due to their high sensitivity and rapid detection. The extracellular ATP is usually measured in the cell supernatants or in the cell cultures by using the standard bioluminescence luciferine/luciferase assay [118]. In contrast, the intracellular ATP is determined using live cell ATP assays that can penetrate the cell membrane without interfering with cellular metabolism or cell integrity, and the luciferase activity is proportional to the intracellular ATP concentration [122]. A major advantage of ATP monitoring is that it does not require culturing microbes for a lengthy period and can provide rapid test results. Under most conditions, the ATP test results correlate well with traditional culture-based test results [126].

## 4. Rapid Chloramine Decay and Nitrification Control Measures

Many studies have suggested that rapid chloramine decay and nitrification can be minimised by maintaining a minimum chloramine residual concentration of 2 to 3 mg ${\mathrm{Cl}}_{2}{\;L}^{-1}$ [7,10,17,44,127]. However, maintaining such a chloramine residual at the farthest part of the distribution system may not be always possible, as it will require a significant increase of chloramine residual concentration at the nearest part of the distribution system. Some utilities apply re-chlorination at certain intermediate points of the distribution system to boost the chloramine residual. It should be noted that uncontrolled blending of chlorinated and chloraminated water can occur near the re-chlorination point and may cause an increase in DBP levels or a decrease in chloramine residual or breakpoint chlorination. Once nitrification is established in a drinking water system, often it is difficult to control by increasing the chloramine residual level, even up to 8 mg ${\mathrm{Cl}}_{2}{\;L}^{-1}$ [41,98]. This suggests that a large portion of chloramine residual is lost through the reaction with nitrification products, before it starts inactivating nitrifying bacteria and SMPs [7,17,40]. Moreover, during the stages of rapid chloramine decay and nitrification, there is an additional chlorine demand by SMPs that needs to be fulfilled to effectively recover the chloramine residual in the system after re-chloramination [128].

pH can affect the nitrification process in many ways, such as by affecting the rate of chloramine auto-decomposition, the rate of ammonia release from chloramine decay, and growth and inactivation rates of nitrifiers by chloramine [12,44]. Waters with a high pH, usually above 8.5, can reduce the chloramine decomposition rate, hence, decreasing the free ammonia formation. For a drop of pH of 0.7 units, the rate of chloramine decay approximately doubles [129]. Some studies reported to reduce the nitrification frequency by raising the pH level to 9 or above [7,130]. An elevated pH level also reduces the dichloramine formation, which has a bad taste and odour and may cause customer complaints [7,130]. The optimal pH for AOB and NOB growths are 7.5 and 7, respectively [131]. However, other studies suggested different optimal pH values for AOB and NOB activities, ranging between 7.9 to 8.6 pH units [132,133]. The ammonium oxidation can be completely inhibited at a pH of 5, while the nitrite oxidation is strongly inhibited at a pH of 8.5 [131]. pH also affects the nitrification process by shifting the acid–base equilibriums, hence, affecting the substrate availability required for AOB and NOB activities [133]. It has been shown that raising the pH level reduces lead and copper leaching to the water, thereby improving corrosion control [12]. However, a high pH value can decrease the inactivation rate of AOB by chloramine [44]. Therefore, controlling nitrification by adjusting pH is often site-specific, and utilities should decide the pH level based on their regular experiences. Although an elevated level of pH can significantly improve the control of nitrification, it may not completely eliminate the problem [130]. Hence, other measures, such as minimising excess ammonia in the distribution system or flushing the system at regular intervals, should be applied together with pH adjustment.

**Figure 4:**
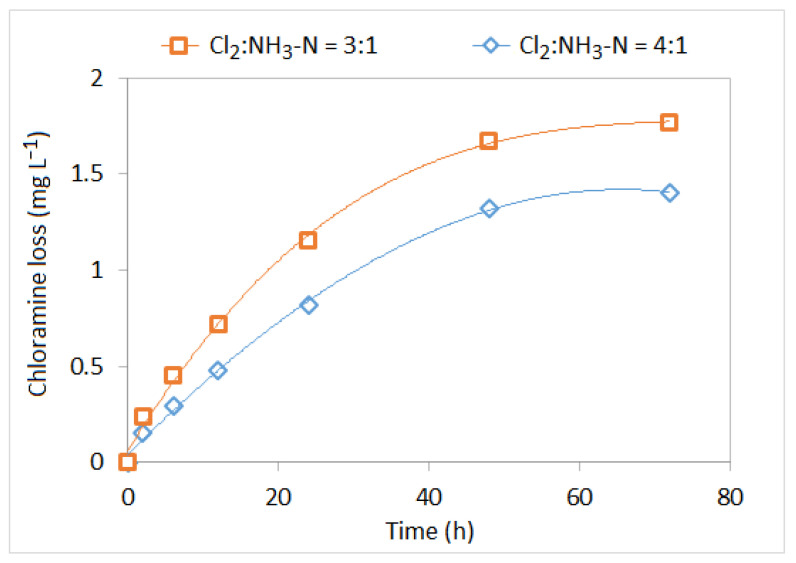
Chloramine decay at chlorine-to-ammonia ratios of 3:1 and 4:1 with initial chloramine concentration = 3 mg $L^{-1}$, TOC = 2.6 mg $L^{-1}$, pH = 7.0, and T = 25 °C (based in part on Zhou et al. [134], Water Environment Research © 2013 Wiley).

Optimising the chlorine-to-ammonia ratio is also shown to be effective in controlling nitrification [2,45]. A survey by Seidel, et al. [2] reported that this strategy is adopted to control nitrification in 68% of cases. In fact, a relatively high chlorine-to-ammonia ratio can ensure a lesser amount of free ammonia available to promote nitrification. Free ammonia can be significantly reduced at a chlorine-to-ammonia ratio of 5:1 [7]. Typically, during monochloramine formation, most water utilities apply a chlorine-to-ammonia ratio that varies from 3:1 to 5:1 [1]. As shown in Figure 4, Zhou et al. [134] reported that chloramine is more stable at a chlorine-to-ammonia ratio of 4:1 compared to 3:1. For a chlorine-to-ammonia ratio of 4:1, chloramine decay rate was 0.0196 mg $L^{-1}{\;h}^{-1}$, while the same decay rate was 0.0246 mg $L^{-1}{\;h}^{-1}$ when the ratio was 3:1 [134]. According to Liu, et al. [115], nitrification can be minimised by maintaining a combined chlorine residual of 1 mg $L^{-1}$ or above, with a chlorine-to-ammonia ratio of 5:1. Fleming, et al. [98], defined a threshold chlorine value of 1.6 mg $L^{-1}$ or above that should be effective to minimise the nitrification risk. When the chlorine concentration drops below 1.6 mg $L^{-1}$, the nitrification potential greatly depends on the chlorine-to-ammonia ratio [45]. By assessing the slope of the nitrification potential curve, Zhang, et al. [12] suggested that nitrification can be prevented when the biocide (chlorine)-to-food (ammonia) mass ratio is eight or above. Harrington, et al. [44] observed that nitrification did not happen when the total chlorine residual was 2.2 mg $L^{-1}$ or above and the biocide-to-food ratio was 1.9 mg ${\mathrm{Cl}}_{2}{\;\mathrm{mg}}^{-1}$ of N or above. Similar studies also confirmed that nitrification can be significantly reduced by changing the chlorine-to-ammonia ratio from 3:1 to 5:1 [40,45]. This is also evident in PDS, where nitrification was found to occur at a chlorine-to-ammonia ratio of 3:1 [45]. A study by Shi, et al. [28] suggested that nitrification can be minimised by changing the hydraulic regimes and disinfection scenarios. They found that nitrification becomes severe when the fluid flow transforms from laminar to turbulent (2300 < Re < 4000). Optimising the chlorine-to-ammonia ratio and increasing disinfectant concentration can inhibit nitrification to some extent under turbulent flow conditions [28].

A theoretical concept developed by the American Water Works Association (AWWA) [64] describes nitrite/nitrate production based on chloramine decay stoichiometry as a function of chlorine-to-ammonia-N ratio. Theoretically, a maximum contaminant level of nitrite-N (1 mg $L^{-1}$) could be exceeded when the chloramine dose is approximately 3 mg $L^{-1}$ (as total ${\mathrm{Cl}}_{2}$) or above and chlorine-to-ammonia-N ratio is less than 5:1. Organic oxidation by chloramine (Table 1) was considered as the reaction pathway, with 100% decay, as well as 100% conversion of ammonia to nitrate/nitrite-N being assumed. Their analysis indicate that nitrite/nitrate production can be minimised by increasing the chlorine-to-ammonia-N ratio. In contrast, Zhang et al. [135] suggested that TOC level has a higher impact on the occurrence of nitrification, while chlorine-to-ammonia ratio has little or no significant impact on nitrification occurrence. According to Lieu et al. [127], the easiest and cost-effective way to control nitrification is by maintaining a certain level of chloramine residual throughout the distribution system and optimising the chlorine-to-ammonia ratio during the formation of monochloramine. However, formation of monochloramine at a chlorine-to-ammonia ratio of 5:1 is not always easy and sometimes it is associated with significant amount of dichloramine formation that has a bad taste and odour and often leads to more DBP formation [46]. For instance, an increased level of dichloramine may increase the formation of nitriles and nitrosamines [57].

**Figure 5:**
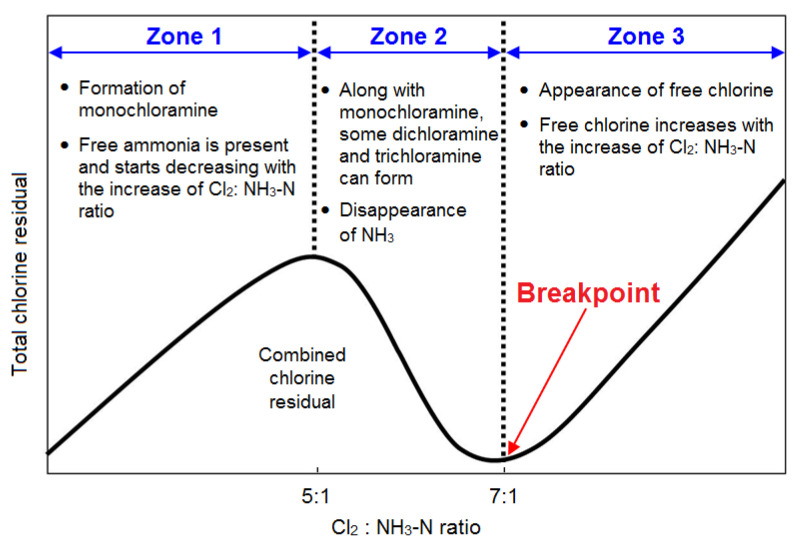
Typical breakpoint curve. Formation of monochloramine dominates in zone 1 with some free ammonia is present; zone 2 is associated with the disappearance of free ammonia and an increased amount of dichloramine and trichloramine formation; free chlorine largely appeared in zone 3.

Another effective control measure for the onset of nitrification is through switching to breakpoint chlorination [17,41]. A survey by Seidel, et al. [2] reported that about a quarter of the surveyed water utilities regularly switch to free chlorine, at least once per year, to prevent nitrification resulting from the seasonal variation of temperature. As shown in Figure 5, the breakpoint residual begins when the rate of chlorine dosage exceeds the demand created by reducing agents, ammonia, and organics. The shape of the breakpoint curve depends on several factors, including pH, temperature, chlorine-to-ammonia ratio, and contact time. A major advantage of applying breakpoint chlorination is that chlorine is largely present in the form of free chlorine and can combine with free ammonia that may be released through chloramine auto-decomposition and/or during the various stages of nitrification. However, breakpoint chlorination may increase the HPC and coliform growth because of chlorine’s poor ability to disinfect particle-associated bacteria [41]. Moreover, chlorine is less capable of penetrating the biofilm to limit the biofilm bacteria [17]. A prolonged use of breakpoint chlorination can make the water have a chlorinous taste, which might cause customer complaints. To minimise the DBP level, breakpoint chlorination should be applied after DBPs’ precursor removal. During nitrification, if a significant level of nitrite is accumulated in water and breakpoint chlorination is applied, nitrogenous DBPs such as nitrosamines and nitramines may form through the reaction of nitrite and hypochlorite [57]. Hence, to minimise the formation of these species, breakpoint chlorination should be applied in such a way that no significant amount of free chlorine residual is left in water.

Chloramine is reactive to NOM, with the rate of oxidation becoming faster as NOM concentration increases. Consequently, chloramine residual level drops, which favours the growth of nitrifying bacteria [12,44,104]. A high level of TOC can promote nitrification in biofilm and bulk water [135]. Therefore, removing a portion of NOM from water during the treatment process can be considered as a long-term nitrification control strategy. Enhanced coagulation is a common practice by WTPs for NOM removal. In a pilot scale study, Harrington, et al. [44] found that NOM removal by enhanced coagulation delayed the onset of nitrification when compared to traditional coagulation methods. More research needs to be done to improve the traditional NOM removal techniques. Similarly, some studies have indicated that chlorite ions can inhibit the ammonia oxidation by *Nitrosomonas europaea* [55,136]. Chlorite ions are produced through the chemical reaction between chlorine dioxide (${\mathrm{ClO}}_{2}$) and water and they are highly reactive in nature. According to Karim and LeChevallier [55], nitrification cannot be effectively controlled by adding up to 0.5 mg $L^{-1}$ of chlorite, especially after long-term application. However, in a pilot study by Rungvetvuthivitaya, et al. [56], they suggested that the direct application of chlorite at a concentration of 0.8 mg $L^{-1}$ can effectively control nitrification. In copper surfaces, application of chlorite at a concentration of 20 ppm can inhibit nitrification, although chlorite can increase copper corrosion [45]. Both chlorine dioxide and chlorite are regulated compounds and their recommended limits in drinking water are 0.4 and 0.8 mg $L^{-1}$, respectively [137]. Consuming concentrations beyond this limit may cause many health issues, including irritation in the mouth and respiratory problems.

Another possible approach to control nitrification is by controlling the nutrient and toxicity levels in water. Some nutrients can boost nitrifying bacterial growth, while some act as inhibitors and slow down the nitrification process. However, many of these substances are toxic in nature, and therefore care must be taken to comply with their regulated limits. Zhang and Edwards [53] found that the nitrification process is strongly inhibited by phosphate levels below 5 μg $L^{-1}$ and copper and zinc levels above 100 μg $L^{-1}$. A relatively high level of phosphate can potentially reduce the concentration of copper and zinc ions and make nitrification more likely. Similarly, a high level of inorganic carbon/alkalinity (>5 mg $L^{-1}$ as ${\mathrm{CaCO}}_{3}$) enhances biomass growth, as they reduce the toxicity of copper and zinc, although nitrifiers require a very low level of inorganic carbon/alkalinity (<5 mg $L^{-1}$ as ${\mathrm{CaCO}}_{3}$) for their growth. A similar study by Sarker, et al. [51] suggested that a combination of zinc and copper strongly reduces the chloramine decay in nitrifying water when compared to zinc application only. Their study found that applying zinc and copper together reduces the chloramine decay rate from 0.0072 to 0.0004 $h^{-1}$. Hence, applying them together can provide better control of rapid chloramine decay and nitrification. In a pilot system study, Lee et al. [49] effectively inhibited nitrification by adding copper. Wagner, et al. [52] observed that adding copper can effectively increase the ammonium removal rates and capacity of biological filters. Copper is found to fully penetrate filters and has the potential to provide long-term nitrification control in biological filters.

Similarly, Bal Krishna and Sathasivan [47] found that adding silver at a concentration of 0.1 mg-Ag $L^{-1}$ is effective in inhibiting the accelerated chemical and microbiological chloramine decay in a laboratory-scale system. The addition of silver was also found to be effective in minimising the part of chemical chloramine decay caused by unknown dissolved compounds in severely nitrified waters. However, their study did not indicate the optimum dose of silver and further study is required to confirm the optimum dose of silver in a real distribution system. Liu et al. [50] and Choi and Hu [48] also found that the abundance of AOB and NOB decreased in the presence of silver nanoparticles. In contrast, some studies found that THMs have the potential to inhibit the nitrification process via the products by cometabolism reactions [110,138]. To prevent nitrification using THMs, the AOB inactivation rate, resulting from by-product toxicity, must be greater than the growth rate. Alternatively, GNPs was found to decrease the nitrification efficiency in an activated sludge system [54]. The application of GNPs potentially reduced the concentration of viable bacteria, including AOB and NOB cells. After dosing with GNPs, the surrounding EPS layer was found to break up from the bacterial cells. Currently, this method has not been practiced in drinking water systems and further research needs to be done to validate this method.

Rapid chloramine decay and nitrification can also be minimised by upgrading the system properties, such as installing recirculation facilities near elevated storage, designing new reservoirs or additional mains to prevent short-circuiting, looping the dead-end mains, and retrofitting reservoirs with baffles to improve the circulation [17]. Operational conditions that lower the water age can minimise nitrification. Designing an adequate mixing system and increasing the daily turnover of water in storage facilities, especially during low water usage periods, can reduce water age [17,40]. When the water usage drops, flushing the mains could be another option to keep new water moving into the system. For short-term nitrification control, flushing has been proven to be effective [17,41], and it has been implemented in about 54% cases to control nitrification [2]. Flushing needs to be done frequently for effective control of nitrification. The bacterial community in biofilm and pipe sediment largely determines the microbial quality of tap water [139]. While flushing is done, a considerable portion of tubercles and sediments are removed allowing disinfectant to reach inside the biofilm matrix, hence, assisting in better control of biofilm bacteria. Ice pigging, which involves pumping ice-slurry into pipes under pressure, also helps to clean up the pipe interior. Moreover, corroding pipes have plenty of crevices, allowing nitrifying bacteria to escape from disinfectant. So, replacing the problematic or old aged components with newer and less corrodible fittings can help to minimise nitrification.

## 5. Summary, Research Gaps, and Recommendations for Future Work

The summaries of the reviewed contents are presented in Table 4 and Table 5.

Over the course of this review, we have identified several research gaps. Firstly, a major concern of water utilities is to minimise the likelihood of nitrification, which has been achieved by regularly performing microbiological tests or monitoring surrogate parameters. However, the majority of these tests require days to weeks to get a conclusive result. An alternative approach, using the optical method, could be an area of interest. Many studies have shown that the UV-vis spectra fingerprints of chloraminated water have a good correlation with organic, chloramine, nitrate, and nitrite. During nitrification, chloramine and organic levels drop, while nitrate and nitrite levels increase, which should be reflected in the nitrified sample spectra. When a sample undergoes nitrification, the UV absorbance within the 200 to 220 nm region (where typical nitrate and nitrite peaks appear) will increase, while the same at 245 nm and 254 nm (where typical monochloramine and organic peak appear) will decrease. Although a method proposed earlier used specific wavelengths sensitive to organic, nitrite, and nitrate absorbances, further refining it by incorporating chloramine absorbance may improve its performance. Possibly, this method can be extended to the real-time monitoring of nitrification in a WDS.

Secondly, although surrogate parameters provide rapid and quick detection of nitrification to some extent, different parameters have different levels of responses to nitrification. Hence, to obtain a clear snapshot of the nitrification status, several parameters need to be evaluated at the same time, rather than using a single parameter. Surrogate parameters detect the effect of water quality resulting from nitrification, but do not represent the actual level of nitrifying bacteria. Using data analytics techniques, it would be possible to develop empirical formulas that are site specific, to relate many of these parameters to the concentration of nitrifying bacteria. This can help form a better assessment of nitrification in a WDS. Moreover, little research has been done about the change of these parameters due to the changes of SMPs during nitrification. Future research can help to better understand this aspect.

Thirdly, some studies have confirmed that the application of silver, zinc, copper, and GNPs can potentially inhibit nitrification. However, most of these studies were conducted in lab-scale or pilot systems. Further investigations are required to confirm their applicability and optimum dosage in real distribution systems. Wall reactions are prominent in real distribution systems, which will affect the nitrification inhibition performance using these methods. Furthermore, health issues associated with long term application of these substances also need to be assessed.

Finally, many methods are available to minimise the likelihood of nitrification, however, the successful outcome is often site-specific. It would be of interest to investigate the potential of applying different methods together to understand their combined effect, or through better analytics to understand different methods in combination, to effectively manage nitrification in a WDS. The nitrifying bacteria distributed in both biofilm and bulk water significantly contribute to the nitrification process. A majority of the nitrification studies were conducted in bulk water. In contrast, the contribution of biofilm bacteria to nitrification is the least-studied area, and further work is required. The likelihood of nitrification can be greatly minimised by controlling the biofilm bacteria. Further research in this area would help us to better understand and adopt appropriate strategies to control nitrification in a WDS.

## 6. Conclusions

A comprehensive review of the current understanding of nitrification, its monitoring, and its control strategies in drinking water systems is presented in this paper. Through nitrification processes, the nitrogen compounds, mainly free ammonia, are oxidised to nitrite and nitrate by nitrifying bacteria. Water utilities that use chloramine as a secondary disinfectant often experience nitrification during the summer season in tropical and/or temperate climates. Nitrification increases nitrite and nitrate levels in water, which, if exceeded beyond the regulated limits, pose threat to public health. Nitrifying bacteria can significantly accelerate the chloramine decomposition by producing soluble microbial products. They can be resistant to disinfectants by creating extracellular polymeric substances surrounding their cell. The best way to monitor the biological activity or nitrification is by performing microbiological tests, such as most probable number, next-generation sequencing, flow cytometry, polymerase chain reaction, fluorescent antibody test, fluorescence in situ hybridisation, and direct counting of bacterial cells. However, some of these techniques are culture-based method that require several days to complete the tests. Alternatively, water utilities use surrogate parameters that indirectly indicate the degree and intensity of nitrification. Some of these parameters include, pH, alkalinity, monochloramine, free ammonia, dissolved oxygen, nitrate, nitrite, and heterotrophic plate count. Additionally, microbial decay factor is increasingly used to monitor rapid chloramine decay and nitrification in WDS.

To control nitrification in a WDS, water utilities adopt various methods, including by controlling various factors that decrease the monochloramine decay rate and limit the ammonium substrate availability. Nitrification likelihood can be minimised by increasing the chloramine residual concentration, decreasing free ammonia concentration, adjusting system pH, optimising the chlorine-to-ammonia ratio, re-chlorination at intermediate points, switching to breakpoint chlorination, and decreasing the NOM level in treated waters. Moreover, a few studies have suggested that the use of zinc (Zn), copper (Cu), silver (Ag), chlorite ion (${\mathrm{ClO}}_{2}^{-}$), and graphite nanoparticles potentially inhibit nitrification. Among the available methods, some show good performance in a WDS, while showing poor performance in a different WDS, because the treated water characteristics among the water utilities vary.

The key findings in this review are: (i) the performance of some methods can be different in the case of a real WDS, as the actual studies were conducted in a laboratory or on pilot-scale systems; (ii) there are improved methods/tools that are required to adequately provide early warning of nitrification; (iii) for surrogate parameters, although helpful in assessing the nitrification status, there is no relationship found between them with the concentration of nitrifying bacteria; (iv) further investigations are required to better understand the role of soluble microbial products on the change of surrogate parameters. This review should not be considered as a detailed and broad review, covering all aspects in this area; rather it reviews the major methods/strategies to monitor and control nitrification in a WDS. It is mostly based on peer-reviewed published articles. Future studies should consider grey literature as well to ensure all existing research in this area is included, to address gaps in the current study. Based on the current review, we recommend that the best method to monitor and control nitrification in a WDS is often site-specific and should be determined in line with utilities’ individual experience, with considering the sustainability and economic aspects.

## Figures and Tables

**Table 1:** Chemical reactions that govern the release of ammonia in the distribution system.

Reactions Involved during Ammonia Release	Reaction Description
$3{\mathrm{NH}}_{2}\mathrm{Cl}\to N_{2}+{\mathrm{NH}}_{3}+3{\mathrm{Cl}}^{-}+3H^{+}$	Chloramine auto-decomposition
$\frac{1}{10}C_{5}H_{7}O_{2}N+{\mathrm{NH}}_{2}\mathrm{Cl}+\frac{9}{10}H_{2}O\to \frac{4}{10}{\mathrm{CO}}_{2}+\frac{1}{10}{\mathrm{HCO}}_{3}^{-}+\frac{11}{10}{\mathrm{NH}}_{4}^{+}+{\mathrm{Cl}}^{-}$	NOM oxidation by chloramine
$\frac{1}{2}{\mathrm{NH}}_{2}\mathrm{Cl}+H^{+}+{\mathrm{Fe}}^{2+}\to {\mathrm{Fe}}^{3+}+\frac{1}{2}{\mathrm{NH}}_{4}^{+}+\frac{1}{2}{\mathrm{Cl}}^{-}$	Reaction of chloramine with pipe corrosion products
$3{\mathrm{NH}}_{2}\mathrm{Cl}\to N_{2}+{\mathrm{NH}}_{3}+3{\mathrm{Cl}}^{-}+3H^{+}$	Chloramine catalysis reactions at pipe surface
${\mathrm{NH}}_{2}\mathrm{Cl}+{\mathrm{NO}}_{2}+H_{2}O\to {\mathrm{NH}}_{3}+{\mathrm{NO}}_{3}+\mathrm{HCl}$	Nitrite oxidation by chloramine

**Table 2:** Nitrification indicators.

Parameters	Symptoms	Description
pH	Decrease	Ammonia oxidation by nitrifying bacteria produce acid which may drop the system’s pH. However, in most drinking water system, a significant change of pH due to microbiological activity is not observed [9].
DO	Decrease	Nitrifying bacteria are aerobic type organisms, consuming oxygen from water resulting in a reduction of DO level in water. Although, interaction between various chemical species such as corrosion reactions may reduce the DO level, so it cannot be used as a standard indicator for rapid microbiological decay and nitrification.
Alkalinity	Decrease	Nitrifiers use alkalinity as carbon source to produce more cell mass. This way they consume a large amount of alkalinity. However, a significant reduction of alkalinity due to microbiological activity is not found in most drinking water systems [9]
Monochloramine	Decrease	Ammonia oxidation by AOB produces nitrite which is reactive to monochloramine. Consequently, its decay rate becomes faster due to the chemical reaction with nitrite [69,104]. Moreover, ammonia oxidation may shift the equilibrium of monochloramine formation so that monochloramine is hydrolysed as free ammonia is metabolised [16].
Free ammonia	Initially increase then decrease	As monochloramine decays to free ammonia and chlorine, free ammonia concentration will initially increase then decrease, because nitrifying bacteria consume ammonia for their survival.
Nitrite Nitrate	Increase Increase	Nitrifying bacteria oxidise ammonia to nitrite and nitrate resulting in an elevated level of nitrite and nitrate concentrations in water.
Heterotrophic bacterial growth	Increase	During the various stages of nitrification, the heterotrophic bacterial population increases by several orders of magnitude.
Customer turbidity complaints	Increase	Sloughing-off of biofilm increases customer turbidity complaints. In addition, nitrification can stimulate the corrosion reaction by shifting the redox potential, resulting in red water due to iron release [2].
Pipe flow	Increase	As biofilm develops in pipes, it reduces pipe inner diameter resulting in an increased flow velocity in pipes.
UV absorbance within 200 nm to 220 nm	Increase	During nitrification, nitrite and nitrate levels increase, hence, the UV absorbance within 200 nm to 220 nm (where typical nitrite and nitrate peak appear) will increase.
UV absorbance at 254 nm	Decrease	Because of organic consumption by nitrifying bacteria, the UV absorbance at 254 nm (where typical organic peak appears) will decrease.
UV absorbance at 245 nm	Decrease	Typical UV absorbance peak of monochloramine appears at 245 nm, which will decrease due to monochloramine decay.

**Table 3:** Values of microbial decay factor at various stages of nitrification (Source: South Australian Water Corporation’s internal document, based on observed chloramine decay of WTP product and distribution samples over several years).

Status of Nitrification	Monochloramine Decay Status	Water Quality Indicators
Clear	Monochloramine decay is stable and is subjected to chemical factors only.	$F_{m}$ ≤ 0.2 Monochloramine and free ammonia concentrations are stable with no change in oxidised nitrogen concentration.
Mild	Monochloramine decay starts increasing due to the increased microbiological activity.	$\approx 0.3\;<\;F_{m}$ ≤ 1.5 Monochloramine and free ammonia concentrations become stable and possibly no significant change in oxidised nitrogen concentration.
Severe	Monochloramine rapidly decays due to the enhanced growth of microbes, the decay rate further accelerates because of the production of SMPs.	$F_{m}$ ≥ 1.5 Monochloramine and free ammonia concentrations decrease and oxidised nitrogen concentration increases.

**Table 4:** Summary of nitrification monitoring strategies in DWDS.

Monitoring Strategy	Description	Advantage	Disadvantage	References
Microbiological Tests				
Most Probable Number (MPN)	Statistical method to estimate the concentration of viable bacteria in a sample	Simple and widely adopted method to classify a range of bacteria including nitrifying bacteria	Requires a lengthy incubation period of 3 to 15 weeks	[10,40,98]
Fluorescence in situ hybridisation (FISH)	Molecular technique to detect 16S rDNA sequences using a fluorescent probe	High sensitivity and specificity in identifying target DNA sequence	Inefficient to process multiple samples in parallel as bacterial cells are fixed to microscope slides	[33,92,93,99]
Next generation sequencing (NGS)	Massively parallel sequencing of nucleotides in entire genomes or the target regions of DNA (16S rDNA)	Provides ultra-high throughput, scalability, and speed	Generates huge amount of data that require expert analysis to obtain a conclusive result	[35,38,39]
Flow cytometry method (FCM)	Identification and quantitative measurement of particles or bacteria by analysing the optical signal	Rapid, reproducible, and robust method, can characterise individual cells in a population	Not suitable for analysing cells that tend to stick together	[34,36,37]
Polymerase chain reaction (PCR)	Amplifies the target region of the DNA (16S rDNA and *amoA* gene) up to a factor by $10^{9}$ with a high level of accuracy	Easy to understand and use, highly sensitive and can provide rapid test results	Detects nucleic acid components only and does not consider whether they come from viable cells	[31,32,67,100]
Fluorescent antibody (FA)	Fluorescent chemicals are used to visualise the target antigen under a fluorescent microscope	Suitable to visualise multiple cell types using different dyes	FAs can have non-specific binding to EPS	[46,101]
Cell mass counting	Counting the detectable bacterial cell numbers under a microscope using a counting chamber	Simple method and can provide rapid test results, suitable to count very dense bacterial population if they are diluted appropriately	Counts the total cell numbers and does not distinguish between live-cell and dead-cell	[9,32,102,103]
Surrogate Parameters				
pH	Oxidation reaction by nitrifying bacteria produce acids which change the system pH	Simple, portable, cost effective and provide rapid test results	In most WDS, there is no significant change of pH level found during nitrification	[7,9,105]
DO	Nitrifying bacteria consume oxygen resulting in a reduction of DO level in water	Simple, portable, cost effective, and provides rapid test results	Interaction between various species including corrosion reactions may also change the DO level	[7,9]
Alkalinity	Nitrifying bacteria consume alkalinity which reduces the alkalinity of water	Can provide rapid test results	Many reasons including corrosion reactions may change the system alkalinity	[7,9]
Monochloramine	Monochloramine residual unexpectedly drops within a short time because of rapid decay	Helps to locate areas of the WDS susceptible to nitrification	Assessing monochloramine only does not proven to be a reliable indicator of nitrification	[7,9]
Free ammonia	Monochloramine auto-decomposition and ammonia oxidation by nitrifying bacteria changes the free ammonia level in water	A reliable indicator and provide early warning for nitrification	Free ammonia level in the WDS may change due to many reasons including operational error at the WTP	[7,9,105]
Nitrite	Ammonia oxidation by AOB and AOA increases nitrite level in water	Most reliable and earliest possible indicator of an onset of nitrification	Instrument/method might sensitive enough to detect low level of nitrite	[7,9,40,105,106]
Nitrate	Nitrite oxidation by NOB increases nitrate level in water	Reliable indicator and provide evidence of complete nitrification	Does not provide early warning of nitrification	[7,9,40]
TOC	Nitrifying bacteria consume carbon resulting in a change of TOC level in water	A high TOC means more likelihood of nitrification, hence, it can only be considered as a potential indicator	A significant change of TOC is not observed during most nitrification cases	[7,9,105]
HPC	Method to determine the number of live and culturable heterotrophic bacteria in a sample	Indicate the potentiality of water for nitrification, so utilities can take corrective actions	This method does not indicate a specific heterotrophic bacteria, rather it indicates total culturable bacteria	[7,9]
Critical threshold residual	A threshold value concept for chloramine concentration beyond which nitrification is not likely to happen	Simple and easy to implement	This is a general indicator, however, in practical, nitrification is found to occur beyond the threshold limit	[10,17,44,98,107]
Customer turbidity complaints	Enhanced microbial population may change the turbidity of water resulting in increased customer complaints	Helps to improve water quality and treatment performance	Cannot be used as a firm indicator as turbidity can change due to many reasons including corrosion	[9]
Pipe flow	Biofilm development in pipes can affect the hydraulic efficiency of a WDS including pipe flow	Simple and easy to implement with real-time monitoring	Can take several months to cause a noticeable change of flow due to biofilm development	[7,9]
Microbial decay factor ($F_{m}$)	Ratio of microbiological chloramine decay coefficient to chemical decay coefficient	Simple and cost-effective way to monitor nitrification	Takes at least 7 days to get the $F_{m}$ value	[6,42,43]
Adenosine Triphosphate (ATP)	ATP works with other enzymes to transfer energy to cells, higher ATP levels indicate greater cell mass	Can provide rapid test results	Difficult to distinguish whether the rise of ATP level is due to slough-off of biofilms or increased bacterial population	[118,120,124]
Other Methods				
Nitrification potential curve	Evaluating the likelihood of nitrification by assessing the AOB growth rate to their inactivation rate	Simple method and easy to implement, require few data such as total chlorine residual and free ammonia concentration	Change of pH, alkalinity, temperature, and DO can affect the performance by this method	[98]
NI	Monitoring nitrification by assessing the ratio of AOB growth rate to their inactivation rate by chloramine and THMs	Regular monitoring of NI value provides early warning of nitrification	Empirical relation between NI and nitrification episode will require for each WDS to obtain a more conclusive result	[110]
CDI	Monitoring nitrification by assessing the ratio of UV light absorbance at 230 nm to that at 254 nm	Evaluating the CDI value helps to identify susceptible spots for nitrification in a WDS	Only used as a general indicator, recommend to investigate further in case of a relatively higher CDI	[111]
Model study	Simulation of chemical and microbiological species using kinetic or water quality model	Can predict nitrification based on other water quality parameters	Accuracy is site specific, requires regular calibration	[112,113,114,115,116]
UV-vis method	Monitoring nitrification by assessing the change of UV spectra with respect to a baseline	Simple method and does not require any massive chemical analysis	Does not indicate the actual level of nitrifying bacteria	[13]
Bioindicator	Classifies nitrified samples by data analysis using 16S rRNA gene dataset	Provides early detection of nitrification as compared to traditional monitoring using surrogate parameters	Difficult to implement in cases of significant changes of water quality	[30]

**Table 5:** Summary of nitrification control strategies in DWDS.

Control Strategy	Description	Advantage	Disadvantage	References
pH adjustment	Decreasing chloramine decay by increasing system pH	Easiest and cost-effective method, provides better corrosion control and reduce lead and copper leaching	A high pH level can potentially decrease the AOB inactivation rate by chloramine	[7,12,44,130]
Increasing chloramine residual concentration	Increasing bacterial inactivation rate by increasing chloramine residual concentration	Provides sufficient level of chloramine residual at downstream of the WDS without re-chlorination at intermediate points	Increased taste and odour issues and may increase the nitrogenous DBPs	[7,10,17,44,127]
Optimising chlorine to ammonia ratio	Reducing the free ammonia concentration by optimising chlorine-to-ammonia ratio so that most ammonia binds with chlorine	Effective in long term nitrification control	Regular evaluation of water quality is required to decide optimum ratio of chlorine to ammonia	[2,7,40,45,64]
Optimisation of NOM removal	Reducing monochloramine decay by reducing NOM concentration in treated water	Effective in long term nitrification control	Increased chemical costs	[12,44,104,135]
Re-chlorination at intermediate points	Free ammonia released through chloramine decomposition is recombined with chlorine	Provides sufficient level of chloramine residual at downstream of the WDS, hence, useful strategy for long-term nitrification control	Uncontrolled blending of chlorinated and chloraminated water may increase the DBP level	[2,7,45,46]
Breakpoint chlorination	Raising the chlorine level to exceed the oxidant demands so that it largely exists in the form of free chlorine to combine with newly generated free ammonia	High efficiency in removing nitrifying bacteria and improves the water quality by removing colour, which is associated with organics	Long-term application may increase DBPs and HPC and coliform growths	[2,17,41]
Regular flushing	Draining all water from the WDS and fill with new water to ensure low water age	Reduces water quality issues and complaints, also helps to reduce tubercles and sediments from pipes	Economically not viable if applied frequently	[17,41]
Ice-pigging	Cleaning the pipe interior by pumping ice-slurry under pressure	Provides better control of biofilm bacteria	Not very practical for old, weak, or large diameter pipes	
Upgrading system properties	Designing improved circulation with adequate mixing system and looping the dead-end mains to reduce water age	Can significantly reduce the likelihood of nitrification	Additional cost may involve with installing and upgrading new system components	[17,40,41]
Controlling hydraulic regime	Affecting nitrification by transferring mass (nutrients and disinfectants) to biofilms, attachment, and detachment of bacteria from biofilm surface	Microbial community and biofilm density vary with hydraulic condition, hence, assist in controlling biofilm bacteria to some extent	Depending on customer water demand, it may not always be possible to maintain the required hydraulic condition	[28,86]
Adjusting nutrient levels	Reducing nutrient levels to limit the nitrifiers growth or increasing nutrient limit to inhibit the same growth	Nutrients such as phosphate at low levels can inhibit nitrification while high levels reduce biofilm cell numbers and helps to control corrosion and metal leaching	High phosphate levels can reduce toxic metals concentration and make nitrification more likely	[53]
Zinc	Zinc nanoparticles (Zn-NPs) can inhibit nitrification by destroying the integrity of cell membranes	Zinc concentration within the regulated limit can potentially reduce the chloramine decay rate	Low levels of zinc can promote the growth of some pathogens	[51]
Copper	Inhibiting microbial growth by its toxicity, causing rapid loss of membrane integrity	Increases ammonium removal rates from biological filters, copper can fully penetrate the filter, hence, provides better control of microbes in biological filters	Maintaining the desired level of copper to inhibit nitrification at the farthest point of the WDS may not be possible because of its accumulation in pipes	[49,51,52]
Silver	Antibacterial property of silver slows down the enzymatic function and promotes membrane permeability resulting in disrupting the membrane integrity	Silver nanoparticles (Ag-NPs) have long-lasting effect and enhanced bactericidal activity that helps to minimise nitrification	Interaction of Ag-NPs and nitrifying bacteria may stimulate the $N_{2}O$ emission	[47,48,50]
Graphite nanoparticles (GNPs)	Decreasing nitrification efficiency by stronger cytotoxic effects of GNPs on the nitrifying bacteria	Disappearance of EPS layer surrounding the bacteria cells, hence, assists in controlling biofilm formation	Some microorganisms show greater stability during GNPs treatment	[54]
Chlorine dioxide and chlorite ion	Chlorine dioxide and its reduction by-product, chlorite ions, can inhibit the formation of AOB, NOB, and biofilms	Effective against EPS layer, hence, provides better control of biofilm bacteria	Can potentially increase corrosion in metallic pipes	[45,55,56,136]
THMs	AOB can biodegrade THMs and produce cometabolism by-products which are highly reactive and can kill or damage AOB cells	THMs’ cometabolism could play a significant role to inhibit nitrification in parts of the WDS where disinfectant concentration is low	Potentially toxic, hence, long-term application of THMSs may cause health issues	[110,138]

## Data Availability

All data sources are cited in the manuscript. Please refer to the cited sources or contact the authors for further information.
